# Sustainability assessment of hybrid active greenhouse solar dryer integrated with evacuated solar collector

**DOI:** 10.1016/j.crfs.2021.09.011

**Published:** 2021-10-02

**Authors:** Pushpendra Singh, M.K. Gaur

**Affiliations:** Department of Mechanical Engineering, Madhav Institute of Technology and Science, Gwalior, India-474005

**Keywords:** Greenhouse, Dryer, Solar, Tomato, Economic

## Abstract

Environment and Economy are the two important pillars of sustainability. In this paper, the economic viability and environmental impact of the novel greenhouse dryer with an evacuated solar collector are calculated. For this analysis, tomato is dried inside the dryer as it is a high moisture crop that requires a faster drying rate otherwise it starts giving a bad odor and gets contaminated. The hybrid active greenhouse dryer is developed especially for drying high moisture agro and non-agro-based produce. Evacuated tube solar collector is integrated with the dryer that supplies the hot water to the heat exchanger kept inside the dryer. The hot water flowing inside the copper tubes of the heat exchanger transfers its heat to room air through convection and to crop through conduction. Hence the higher room temperature and faster moisture removal rate are obtained. Tomato slices have been dried from 94.6% (wb) to 10% (wb) moisture content in 10 h. The developed dryer can produce 261 kg of dried tomato annually and its payback time is only 1.73 years which is very less as compared to its life of 30 years. In its entire lifetime, the dryer will mitigate 169.10 tonnes of CO_2_ that prove its suitability from a sustainable point of view.

## Highlights

• A Novel hybrid greenhouse Solar dryer is developed for drying high moisture crops.
• Dryer is totally dependent on renewable energy source with an increased drying period per day even after sunset.
• The economic analysis shows that the capital cost of hybrid dryer is not too high as compared to other hybrid dryer.

## Graphical abstract

**Figure:**
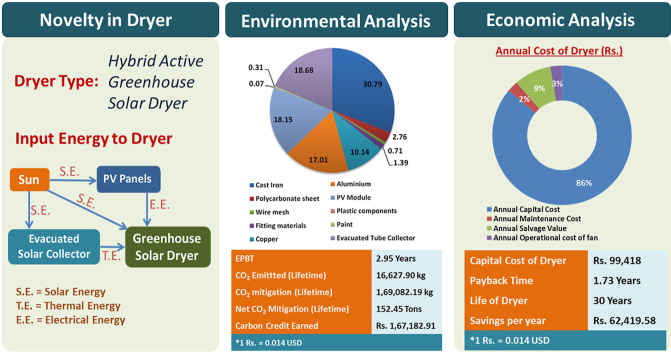

## 1 Introduction

Sustainable development is an effort to fulfil the people's requirement without harming the environment. There must be an ecological balance to maintain the existence of life on Earth. Any developed product should not harm the environment in any way during its lifetime. Solar dryers are developed as a sustainable solution to reduce the scarcity of food by drying it and storing it for a long duration. As the solar dryers are operated by renewable source of energy hence it does not harm the environment. Solar dryers were basically developed to dry the crops to safe moisture levels by using a renewable energy source i.e. sun. The solar dryers are not limited for agricultural purposes but it is also used in industrial purposes for process heating (Abdulmalek et al., 2018; Boonyasri et al., 2011).

The greenhouse is one of the inventions in field of solar drying that harnesses the solar energy for space heating, drying, or agricultural purpose (Kumar et al., 2016). As solar energy is one of the renewable energy sources in which very faster innovations and developments are taking place. For making the greenhouse more efficient, these are operated mostly in active mode. In an active mode, the electric power required to operate the fans is powered by PV panels (Eltawil et al., 2018a; Chauhan et al., 2018a). By 2020, mixed mode dryers can reduce CO_2_ emissions by 23% (Tripathy, 2015). The energy consumption from the conventional sources can be reduced by 27–80% by using 40% efficient solar dryers (Tripathy, 2015; Arata et al., 1993).

The greenhouse drying is a sustainable method of drying products as it is low cost and energy saving (Kumar et al., 2021a). The greenhouse dryers are now made hybrid to dry the high moisture crops as these require more energy to maintain the faster drying (Shaikh and Kolekar, 2015). The hybrid dryers can also be operated in the off sunshine period by storing the excess energy supplied by a secondary sources like biomass, LPG, or solar energy used separately in solar water heaters, etc. (Abdulmalek et al., 2018; Janjai, 2012; Khanlari et al., 2020). The use of some additional device in the hybrid dryer makes it a little bit costly but the extra amount invested on it can be compensated by faster drying means reduced drying time per batch. Hence more crops can be dried annually and that will give higher annual savings from the dryer.

The cost and the environmental impact are some major criteria that decide the feasibility of a developed product for commercial scale and also decide its suitability from a sustainable point of view. Various researches had been done on the economic analysis of hybrid solar dryers (Mustapha et al., 2014; Ndukwu et al., 2020; ELkhadraoui et al., 2015; Selvanayaki et al., 2017). Boonyasri et al. (2011) carried out the economic analysis of forced mode greenhouse solar dryer for drying pork. The payback time of the dryer was 1.15 years and the capital cost was 49,500 Baht. Hamdani et al. (Hamdani and Muhammad, 2018) carried out the economic analysis of biomass integrated hybrid dryer. The Break-even point for the developed dryer was 2.6 years with an NPV of $21.091. Dhanushkodi et al. (2015) carried out the life cycle cost analysis of a hybrid dryer for drying cashew nuts. The experimentation was carried out on solar, biomass, and hybrid drying system and the payback-time was 1.58, 1.32, and 1.99 years respectively. Prakash et al. (2016) developed the modified greenhouse dryer with thermal storage material on the floor of the dryer. The payback time was 1.11 years and 1.89 years while the capital cost was Rs. 8994.50 and Rs. 12844.50 for passive and active mode. Kaewkiew et al. (2012) developed the semi-cylindrical greenhouse dryer whose payback time for the dryer was about 2 years which is very less from a commercial point of view.

In the study on the mixed mode solar dryer, the maximum CO_2_ mitigation potential was observed maximum for the replacement of coal with solar energy (Tripathy, 2015) (Singh and Kumar, 2013). Atalay and Cankurtaran (2021) developed the large-scale dryer with thermal storage and dried strawberry inside it to test its performance. The energy payback time of the developed dryer was 6.82 years while it can mitigate 99.60 tons of CO_2_ in its lifetime. Ayyappan (2018) carried out a study on a passive greenhouse dryer with biomass heater and dried coconut to evaluate its thermal performance. The dryer mitigates 678 tons of CO_2_ in its lifetime and emits 1.518 tons of CO_2_ annually. Vijayan et al. (2020) carried out the environmental analysis of greenhouse dryers in active and passive mode. Dryer in active mode saves 3.3 tonnes CO_2_ more than the passive mode in a life of 35 years. Kumar et al. (2021b) observed that the cost of greenhouse rises with rise in its embodied energy. Shrivastava and Kumar (2017) dried fenugreek leaves inside the indirect solar dryer and it was observed that the dryer is capable to mitigate 391.52 kg of CO_2_ annually while it emits only 85.46 kg of CO_2_ annually.

From the literature survey it is observed that the greenhouse dryer are made hybrid by using fossil fuel based secondary source which is not a sustainable solution for drying the crops. So this hybrid active greenhouse solar dryer (HAGSD) is developed in which the solar collectors are used to supply the additional thermal energy to the dryer. The entire drying system is stand alone and is operated by solar energy only.

The novelty in this experimental work is the heat exchanger kept inside the greenhouse dryer. The heat exchanger also works as the drying bed for the crop. The hot water from the evacuated tube solar collector is supplied to the copper tubes of the heat exchanger. The heat in hot water inside the copper tubes is partially conducted to the crop in contact with the tube and partially convected to the inside room air. This raises the room air temperature as well as the crop surface temperature and hence the faster evaporation of moisture from the crop surface takes place. No work is reported till now on the sustainability assessment of such kind of HAGSD with evacuated tube collector (ETC) and a heat exchanger. Hence the environmental and economic analysis of the developed setup is carried out to test its sustainability. Tomato is selected as the crop for the analysis as it is a high moisture crop and the developed dryer is suitable for high moisture crops because of high room air temperature and faster drying rate.

## 2 Experimental setup and instrumentation

**Fig. 1:**
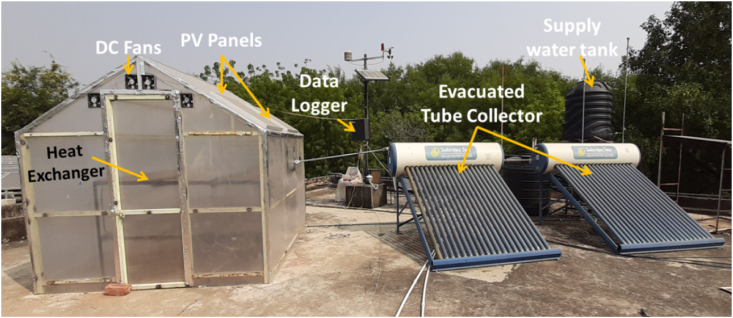
The Pictorial View of developed hybrid active greenhouse solar dryer.

**Fig. 2:**
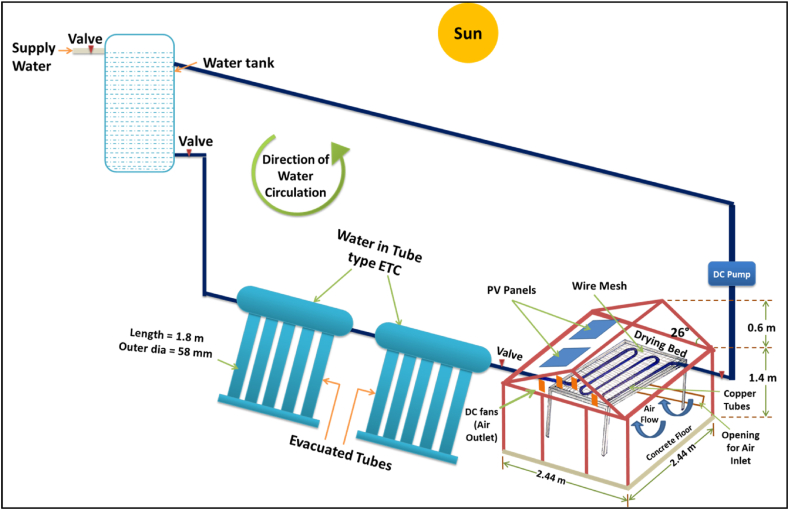
Schematic diagram of developed HAGSD.

The hybrid greenhouse solar dryer has been designed and constructed at Madhav Institute of Technology and Science, Gwalior (26° 14′ N, 78° 10′ E). The frame of the dryer is made of hollow square-shaped iron rods which are fixed at the required position through welding. The joints are properly filled through welding and m-seal is used to make it air-tight as much as possible. The UV stabilized polycarbonate sheet of 6 mm thickness is used as a cover material. Four DC fans were provided to circulate the air inside the dryer. The two PV panels are attached to the roof of the dryer for supplying electricity to operate the fans and DC pump. The hot water from ETC is circulated through copper tubes of the heat exchanger placed inside the dryer using a DC pump. The water in tube type ETC was attached with the heat exchanger placed inside the dryer. The hot water from ETC is supplied inside the dryer through the copper tubes of the heat exchanger bed. The pictorial view and schematic diagram of the HAGSD is shown in Fig. 1 and Fig. 2 respectively.

The opening for air inlet is provided at bottom of the back side of the dryer (West Wall). Four DC fans of rated power 6 W each and having 12 mm and 5 mm as outer and inner diameter respectively are provided at the front to remove the evaporated moisture of crop along with the air inside the dryer. Based on experiment, four fans are found suitable for maintaining the required air circulation inside the dryer. As the ambient air enters from the bottom of the dryer and after heating it rises upward due to buoyancy effect, hence the fans are provided at the top of the front wall. The heat exchanger kept inside the dryer has a dimension as 2 m × 2 m x 1 m. Total 18 U shaped copper tubes joined together are used in heat exchanger to carry the hot water through it.

For measuring the temperature, K-type thermocouples of accuracy ±0.1 °C are placed at a different location inside the dryer. Pyranometer of Central Electronics Limited with ±1 W/m^2^ accuracy is used to measure the solar irradiance. Digital weight balance is used to measure the weight of the crop at the 1-h interval. The Datataker DT85 data logger is used to record and display the reading of measured temperature and solar radiation. The possible uncertainty in the measured parameters is shown in Table 1.

**Table 1:** Uncertainty in measured parameters.

Measured Parameters	Uncertainty
Temperature	±0.275 °C
Weight of crop	±0.269 kg
Solar Radiation	±5.77 W/m^2^

Tomato is one of the most consumed crops in the world that is used for making various dishes. It is also a very high moisture crop so for storage it must be dried to a safe moisture level. For the experiment, the tomato is purchased from the local market and washed properly with fresh water. The tomato is cut into slices of 5 mm thickness and then spread evenly on the drying tray inside the greenhouse. The dryer is operated from 10 a.m. to 8 p.m. as due to the use of heat exchanger inside the dryer, it continues to operate in off sunshine period also till the heat stored in the water of ETC not lost to the surrounding.

## 3 Environmental analysis of HAGSD

### 3.1 Embodied energy

The total energy required to make the complete setup of the greenhouse solar dryer is termed as embodied energy of dryer (E_em_) (Eltawil et al., 2018b; Prakash and Kumar, 2014). The different materials used to construct the HAGSD are given in Table 2 with their corresponding embodied energy (Vijayan et al., 2020; Chauhan et al., 2018b; Barnwal and Tiwari, 2008; Tiwari and Barnwal, 2008) and quantity of material used.

**Table 2:** Embodied energy of different material used to construct HAGSD.

S. No.	Materials	Embodied Energy	Quantity	Total Embodied Energy
		(kWh/kg)	(kg)	(kWh)
**1.**	Hollow Cast Iron Pipe for frame	8.89	207	1840.23
**2.**	Hollow Cast Iron Pipe for Drying bed	8.89	75	666.75
**3.**	Polycarbonate sheet	10.1974	22	224.34
**4.**	Wire mesh	9.67	6	58.02
**5.**	Fitting materials			
(i) Kundi (Door lock)		55.28	0.2	11.06
(ii) Kabja/Hinges		55.28	1.5	82.92
(iii) Nut bolts with washer		9.67	2	19.34
**6.**	Copper tube for heat exchanger	19.61	42	823.62
**7.**	Aluminium Duct tape for insulation	55.28	0.05	2.76
**8.**	PV Module	739 kWh/module	2 Nos.	1478.00
**9.**	Plastic components like DC fan and DC motor casing etc.	19.44	0.3	5.83
**10.**	Copper wire used for electricity supply	19.61	0.1	1.96
**11.**	Paint	25.11	1	25.11
**12.**	Evacuated Tube Collector	422.5 kWh/m^2^	3.6 m^2^	1521.00
**13.**	Aluminium tube with T and L joints	55.28	25	1382.00
**Total Embodied Energy of the HGSD**				**8311.95 kWh**

### 3.2 Energy payback time (EPBT)

EPBT is the amount of time taken by the hybrid dryer to recover the energy invested in it during its construction. Mathematically it is expressed as (Eltawil et al., 2018b; Kesavan et al., 2019).

(1) $$Energy\;Payback\;Time=\frac{Embodied\;Energy\;\left(kWh\right)}{Annual\;energy\;output\;\left(kWh/yr\right)}$$

The annual energy output (E_a_) is calculated using Eq. (2) (Vijayan et al., 2020):

(2) $$E_{a}=Daily\;thermal\;output\times No.\;of\;sunshine\;days\;in\;a\;year$$

Generally, the number of sunshine days in a year is taken as about 290 days in India but it may vary from region to region and climatic condition of the place. The daily thermal output is determined by using the relation given by (Vijayan et al., 2020),

(3) $$Daily\;thermal\;output\;\left(kWh\right)=\frac{Moisture\;Evaporated\;per\;day\;\left(kg\right)\times Latent\;heat\;\left(J/kg\right)}{3.6\times 10^{6}}$$

The value of the latent heat of evaporation of water is taken as 2430 kJ/kg.

### 3.3 Carbon dioxide emitted by the HAGSD

Considering that the electricity is generated using coal then the CO_2_ emission is taken as approximately 0.98 kg/kWh. The annual CO_2_ emission is given by Eq. (4) (Prakash et al., 2016).

(4) $$CO_{2}\;Emission\;per\;year=\frac{Embodied\;Energy\times 0.98}{Life\;of\;dryer}$$

There are various losses related to electricity generation and transmission. If the domestic appliance losses (L_da_) and transmission and distribution losses (L_dt_) are considered then Eq. (4) becomes,

(5) $$CO_{2}\;Emission\;per\;year=\frac{1}{1-L_{da}}\times \frac{1}{1-L_{dt}}\times \frac{Embodied\;Energy\times 0.98}{Life\;of\;dryer}$$

Generally, the value of L_da_ and L_dt_ is taken as 0.20 and 0.40 respectively then Eq. (5) is given as:

(6) $$CO_{2}\;Emission\;per\;year=\frac{Embodied\;Energy}{Life\;of\;dryer}\times 2.042\;,\;kg$$

### 3.4 CO_2_ mitigation by HAGSD

The CO_2_ mitigation per kWh of the greenhouse dryer is given by Eq. (7) (Prakash and Kumar, 2014).

(7) $${\text{CO}}_{2}\text{ mitigation}/\text{KWh}\left(\text{Y}\right)=\frac{1}{1-L_{a}}\times \frac{1}{1-L_{td}}\times 0.98=2.042\;kg/kWh$$

If the CO_2_ mitigation is considered for the entire life of the dryer, then it is given by Eq. (8) as,

(8) $${\text{CO}}_{2}\text{ mitigation }\left(\text{lifetime}\right)=\text{ Embodied Enegy}\times 2.042,\text{ kg}$$

The net CO_2_ mitigated by greenhouse during its entire lifetime is given by Eq. (9).

$$\text{Net Mitigation over lifetime}={\text{Total CO}}_{2}\text{ mitigation}-{\text{Total CO}}_{2}\text{ emission}$$

(9) $$∴\;\;\text{Net Mitigation over lifetime}=\left({\text{E}}_{ao}\times \text{L}-\text{Embodied Energy}\right)\times 2.042\;,\;kg$$

where E_ao_ is the annual energy output and L is the life of the dryer which is taken as 30 years. The life of polycarbonate sheet is about 10 years so its need to be replaced after 10 years which is considered as maintenance cost of dryer while the frame of the dryer and heat exchanger is made of iron and these can last for over 30 years. The ETC has a life of about 25 years so in general the life of drying system has been taken as 30 years during the calculations. Also the life of dryer enclosed with polycarbonate sheet is taken as 30 year in various previous research on greenhouse dryers (Villagran et al., 2021; Nayak et al., 2011).

### 3.5 Carbon credit earned by dryer

One ton mitigation of CO_2_ emission is equal to one carbon credit. The carbon credit earned from the HAGSD is calculated using the relation given in Eq. (10) (Tiwari and Tiwari, 2016).

(10) $$Carbon\;Credit\;earned=Net\;{\text{CO}}_{2}mitigation\times Price\;per\;ton\;of\;CO_{2}mitigation$$

## 4 Economic analysis of HAGSD

The annual cost of the Dryer (C_an_) is given as (ELkhadraoui et al., 2015)

(11) $$C_{an}=C_{acp}+C_{mt}-S_{v}+C_{acf}$$

C_acp_ is the annual capital cost and C_acf_ is the annual operational cost of a fan, which are calculated using Eq. (12) and Eq. (13).

(12) $$C_{acp}=C_{cc}F_{cp}$$

(13) $$C_{acf}=N_{f}\times P_{f}\times C_{ue}$$

where C_cc_ is the capital cost of the dryer, N_f_ is the number of an hour the fan run in a year, P_f_ is the rated power consumed by a fan during its operation and C_ue_ is the electricity charge for one unit.

In this case, the daily operation hour of the fan is 8 h and taking 290 days of full sunshine days in a year in this location, then the number of an hour the fan operate in a year is 2320 h. The rated power of one DC fan is 6 W so the rated power of 4 DC fans is 24 W. The charge of one unit of Electricity is taken Rs. 5/kWh.

In this case, annual Maintenance cost (C_mt_) and annual salvage value (S_v_) is taken as 3% and 10% of the annual capital cost of the dryer (Sreekumar et al., 2008).

The capital recovery (F_cp_) is calculated using Eq. (14) as

(14) $$F_{cp}=\frac{d{\left(1+d\right)}^{n}}{{\left(1+d\right)}^{n}-1}$$

where d is the rate of interest on the amount invested for a long time.

**Fig. 3:**
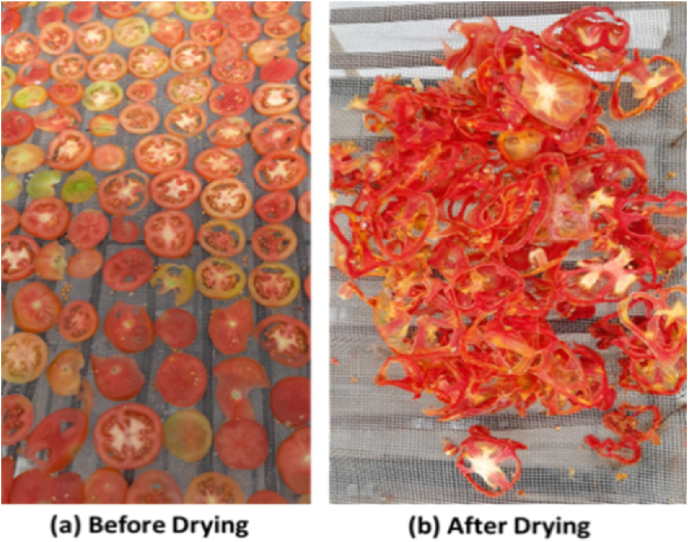
Drying of tomato inside the developed HAGSD.

The total mass of dried tomato in the dryer annually (M_pa_) is given by Eq. (15).

(15) $$M_{pa}=\frac{M_{pd}D}{D_{b}}$$

where M_pd_ is the mass of product dried in a dryer per batch, D is the number of days for which the dryer is used for drying in a year and D_b_ is the number of days taken for drying the material per batch. The capacity of the developed dryer is 15 kg per batch and the drying time of tomato slices per batch is 1 day (10 h). The photograph of the tomato slices before and after drying is shown in Fig. 3.

Then the drying cost for one kg of material (C_u_) is given by,

(16) $$C_{u}=\frac{C_{an}}{M_{pa}}$$

Cost of fresh product per one kg of dried product (C_fd_) is shown by Eq. (17) as,

(17) $$C_{fd}=C_{fp}\times \frac{M_{f}}{M_{pd}}$$

where M_f_ is the mass of fresh product per batch and C_fp_ is the cost of one kg of fresh product.

The cost of one kg of product dried inside the dryer (C_ud_) is given by:

(18) $$C_{ud}=C_{fd}+C_{u}$$

Saving obtained from one kg of product dried inside the dryer (S_kg_) is given by Eq. (19).

(19) $$S_{kg}=SP_{dp}-C_{ud}$$

SP_dp_ is the selling price of dried material for one kg.

Saving obtained from drying one batch is given as,

(20) $$S_{b}=S_{kg}\times M_{pd}$$

Saving obtained from drying of material per day is given by Eq. 21

(21) $$S_{d}=\frac{S_{b}}{D_{b}}$$

The annual saving obtained from the greenhouse dryer in k_th_ year is given by:

(22) $$S_{k}=S_{d}D{\left(1+R_{if}\right)}^{k-1}$$

where, R_if_ is the inflation rate.

Finally, the Payback time (P_b_) is calculated using the relation given by Eq. (23) as follows:

(23) $$P_{b}=\frac{ln\left[1-\frac{C_{cc}}{S_{1}}\left(d-R_{if}\right)\right]}{ln\left(\frac{1+R_{if}}{1+d}\right)}$$

## 5 Result and discussion

**Fig. 4:**
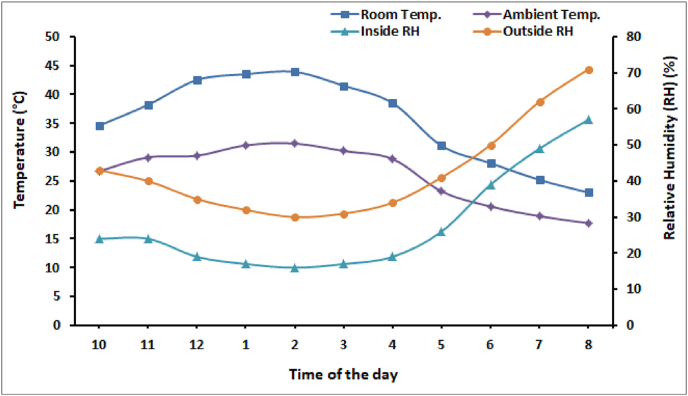
Variation in relative humidity, ambient and room temperature.

The developed hybrid dryer is tested in the winter season on December 3, 2020. The variation in ambient temperature, greenhouse room temperature, inside and outside relative humidity during the experimentation period is shown in Fig. 4. During experiment, the data is recorded on an hourly basis from 10 a.m. to 8 p.m. The ambient temperature ranges from 17.7 °C to 31.5 °C during experimentation time. The room air temperature varies between 23 °C and 43.1 °C. In the winter season also, the dryer rises the inside temperature to a maximum of 13 °C as compared to maximum ambient air temperature.

**Fig. 5:**
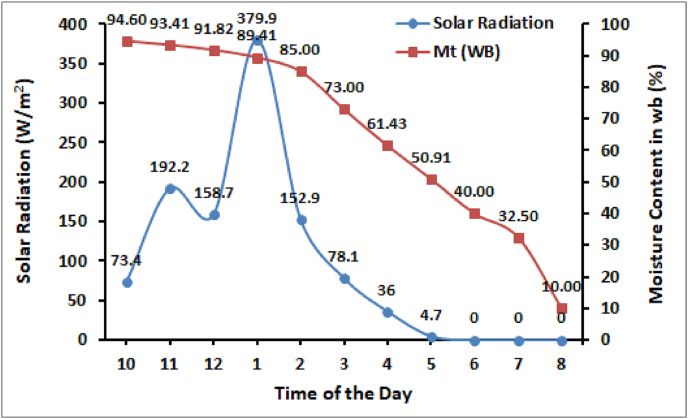
Variation in solar radiation during experimentation period.

As the heat stored in the water of ETC heats the inside air additionally so the room temperature is found sufficiently higher for drying high moisture crops. The variation in relative humidity is found inversely proportional to temperature. Due to high room temperature as compared to ambient temperature, the inside relative humidity is also found lower than the ambient humidity. The maximum value of solar radiation during the experiment was 379.9 W/m^2^. The fluctuations in solar irradiance as shown in Fig. 5 are due to partially cloudy weather during the experimentation period. In the winter season, the intensity of radiation is not as high as in summer and also the ambient temperature is low so the developed dryer is can be used for drying crops, space heating, drying medicinal, and other non-agricultural products in winters.

The initial moisture content of tomato is 94.60% (wb) and it is dried to 10% (wb) moisture level in 10 h as shown in Fig. 5. As the solar radiation increases, the room air temperature also increases which results in a faster drop in moisture content of the tomato slices. With an increase in room temperature, the crop surface temperature also increases, and the diffusion of moisture from inside the crop slice to its surface increases. As the relative humidity is also very low inside the dryer so the water from the crop surface evaporates to the room air fastly and hence the drying rate increases.

### 5.1 Environmental analysis

**Fig. 6:**
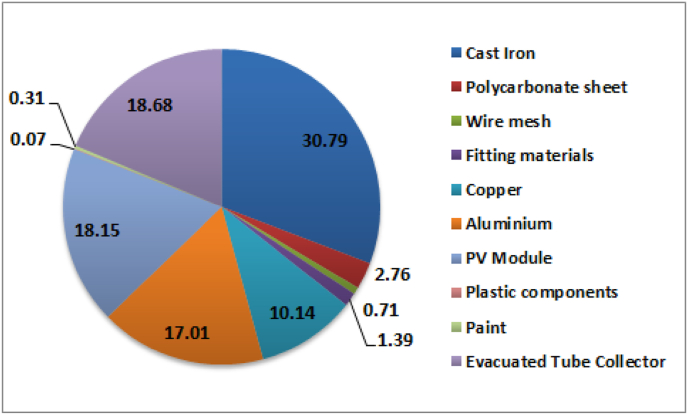
Percentage of Embodied Energy contributed by different material used in the dryer.

The environmental impact of any system depends mostly on its embodied energy. The embodied energy of the materials used in the hybrid dryer is shown in Fig. 6. Cast iron and evacuated tube collector contributes 49.47% together and rest of them is by other materials used in the dryer.

The various environmental parameters calculated to show the impact of the dryer on the environment are shown in Table 4. From the table, it is clear that the energy invested on the dryer can be earned back in a very small time i.e. in 2.95 years only. The total life of the dryer is considered as 30 years and in its total lifetime, the dryer will emit 16.62 tons of CO_2_ while it will mitigate 169.10 tons. This helps a lot in reducing carbon footprint and from the net mitigated CO_2_, about Rs.167,182.91 (Taking 15 USD/tonne of CO_2_ mitigated equivalent to Rs.1096.61/tonne of CO_2_ mitigated) can be earned from the hybrid dryer as a carbon credit.

**Table 4:** Environmental analysis of HAGSD.

Environmental Parameters	Value
EPBT	2.95 Years
Moisture evaporated	14.10 kg/batch
CO_2_ Emittted (Lifetime)	16,627.90 kg
CO_2_ mitigation (Lifetime)	1,69,082.19 kg
Net CO_2_ Mitigation (Lifetime)	152.45 Tons
Carbon Credit Earned	Rs. 1,67,182.91

*1 Rs. = 0.014 USD.

**Fig. 7:**
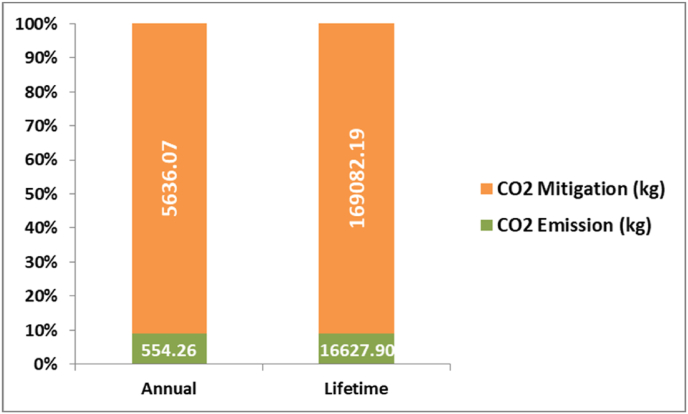
Comparison of CO_2_ emission by developed dryer annually and in its lifetime.

From Fig. 7, it can be depicted that the carbon emission by the developed dryer is very less as compared to carbon mitigated annually as well as in its lifetime operation. This makes the dryer sustainable from an environmental point of view as it can be used for generating heat energy from the use of only solar energy without harming the environment.

### 5.2 Economic analysis

The economic analysis is one of the important factors justifying the feasibility of the developed system. The various economic parameters calculated to evaluate the economic feasibility of HAGSD are shown in Table 5. The capital cost of the dryer is very high due to the inclusion of two evacuated tube solar collectors but the invested amount can be earned back in 1.73 years only, which is very less time as dryer life is 30 years. There is no such annual maintenance is required but still, in the study, we have taken maintenance cost as 5% of the annual capital cost of the dryer.

**Table 5:** Economic analysis of HAGSD.

Cost Parameters	Value	Cost Parameters	Value
Capital Cost of Dryer	Rs. 99,418	Quantity of dried Tomato annually	261 kg
Capital Recovery Factor	9%	Drying Cost of 1 kg of Tomato	Rs. 32.53
Annual Capital Cost	Rs. 8831	Cost of 1 kg fresh tomato	Rs. 45.00
Annual Maintenance Cost	Rs. 264.93	Mass of fresh product per batch (kg)	15 kg
Annual Salvage Value	Rs. 883.10	Cost of fresh product per kg of dried product	Rs. 750.00
Annual Operational cost of fan	Rs. 278.40	Cost of 1 kg tomato dried inside the dryer	Rs. 782.53
Annual Cost of Dryer	Rs. 8491.27	Selling price of 1 kg dried tomato	Rs. 1500
Life of Dryer	30 Years	Saving per kg	Rs. 717.47
Interest Rate	8%	Saving per batch	Rs. 645.72
Inflation Rate	6%	Saving per day	Rs. 215.24
Payback Time	1.73 Years	Saving after 1 year	Rs. 62,419.58
Mass of Tomato dried per batch	0.9 kg		

*1 Rs. = 0.014 USD.

The tomato can be dried and stored for its easier and cheaper availability in all seasons. The developed dryer had a capacity of drying 15 kg per batch and annually it can produce 261 kg of dried tomato. The selling price of dried tomato varies between Rs.1000 to Rs.2000 per kg. In the calculation, it is taken as Rs.1500/kg and this gives the annual saving of Rs. 62,419.58 per day.

**Fig. 8:**
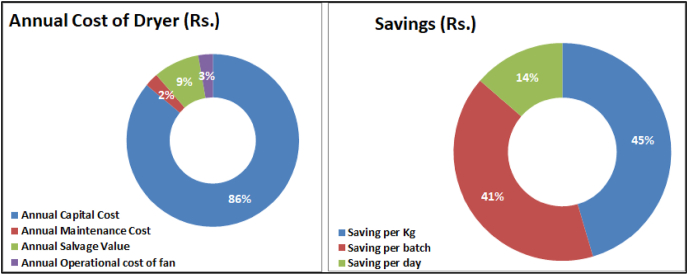
Annual Cost breakup and Savings from developed dryer.

From Fig. 8, it is clear that the annual cost of the dryer is majorly the annual capital cost contributing 86% of the annual cost of the dryer. The annual maintenance and operational cost are very low, which shows that once the dryer is installed there is no major investment required. As the drying time of tomatoes is 2 days, so the per batch saving is a little bit lower than per kg savings as in one batch 0.9 kg dried tomato is obtained during experimentation on 3 kg tomato slices.

The capacity of dryer is 15 kg per batch and drying time is about 2 days, so in a year about 2 Tons of fresh crop can be dried in this developed dryer. The developed dryer is suitable for small-scale industries like the papad industry, cow dung drying in gaushalas, ready-to-make food-making industries, etc. As in conventional open space drying, not only the nutritious value of the dried product is affected but also the drying time is very high which affects the production of any industry. Thus, the developed dryer is not only suitable for large-scale commercial industries but also very helpful for small-scale and rural applications. This shows the economic suitability of dryer from a sustainability point of view.

## 6 Conclusions

The developed HAGSD is a sustainable solution for drying high moisture crops as it is both economically and environmentally feasible. The tomato slices are dried from 94.6% (wb) moisture content to 10% moisture content in 10 h only. The capital cost of the developed hybrid dryer is Rs. 99,418, which is high for small-scale purposes or its use in rural areas. But as the life of the dryer is 30 years and its payback time is 1.73 years only so the invested amount will get recovered soon and hence the profit can be earned for the remaining duration. The maximum temperature difference between ambient and inside room air is 13 °C, which is good for thin-layer drying of high moisture crops in the winter season. The dryer mitigates about 169.10 tons of CO_2_ in its lifetime which will help in reducing the carbon footprints. This makes the dryer a sustainable solution for the drying purpose as it not only reduces carbon emissions but also it is economically viable due to its low payback period. The CO_2_ emission from the dryer depends on its embodied energy, so it can be reduced by using aluminium frames instead of iron frames as it is lighter in weight. The capital cost of the dryer can be minimized for small-scale applications by using only a single evacuated tube collector.

## CRediT authorship contribution statement

**Pushpendra Singh:** Writing – original draft, Conceptualization, Methodology, Writing – review & editing. **M.K. Gaur:** Supervision, Investigation, Visualization.

## Declaration of competing interest

The authors declare that they have no known competing financial interests or personal relationships that could have appeared to influence the work reported in this paper.
